# Identification of a Clinically Relevant Signature for Early Progression in KRAS-Driven Lung Adenocarcinoma

**DOI:** 10.3390/cancers11050600

**Published:** 2019-04-29

**Authors:** Sarah Neidler, Björn Kruspig, Kay Hewit, Tiziana Monteverde, Katarina Gyuraszova, Attila Braun, William Clark, Daniel James, Ann Hedley, Bernhard Nieswandt, Emma Shanks, Craig Dick, Daniel J. Murphy

**Affiliations:** 1 Institute of Cancer Sciences, University of Glasgow, Glasgow G61 1QH, UK; sarahneidler@gmx.de (S.N.); Bjorn.Kruspig@glasgow.ac.uk (B.K.); tiziana.monteverde@manchester.ac.uk (T.M.); Katarina.Gyuraszova@glasgow.ac.uk (K.G.); 2 CRUK Beatson Institute, Garscube Estate, Glasgow G61 1BD, UK; kay.hewit@glasgow.ac.uk (K.H.); w.clark@beatson.gla.ac.uk (W.C.); d.james@beatson.gla.ac.uk (D.J.); a.hedley@beatson.gla.ac.uk (A.H.); e.shanks@beatson.gla.ac.uk (E.S.); 3 Institute of Experimental Biomedicine, University Hospital Wuerzburg, DE 97080 Wuerzburg, Germany; Attila.braun@virchow.uni-wuerzburg.de (A.B.); Bernhard.nieswandt@virchow.uni-wuerzburg.de (B.N.); 4 Rudolf Virchow Center, Julius Maxmilians University of Wuerzburg, DE 97080 Wuerzburg, Germany; 5 Queen Elizabeth University Hospital, Glasgow G51 4TF, UK; Craig.Dick@ggc.scot.nhs.uk

**Keywords:** lung adenocarcinoma, KRAS, MYC, ERBB, Mouse models of cancer, RNA-SEQ

## Abstract

Inducible genetically defined mouse models of cancer uniquely facilitate the investigation of early events in cancer progression, however, there are valid concerns about the ability of such models to faithfully recapitulate human disease. We developed an inducible mouse model of progressive lung adenocarcinoma (LuAd) that combines sporadic activation of oncogenic KRas^G12D^ with modest overexpression of c-MYC (KM model). Histological examination revealed a highly reproducible spontaneous transition from low-grade adenocarcinoma to locally invasive adenocarcinoma within 6 weeks of oncogene activation. Laser-capture microdissection coupled with RNA-SEQ (ribonucleic acid sequencing) was employed to determine transcriptional changes associated with tumour progression. Upregulated genes were triaged for relevance to human LuAd using datasets from Oncomine and cBioportal. Selected genes were validated by RNAi screening in human lung cancer cell lines and examined for association with lung cancer patient overall survival using KMplot.com. Depletion of progression-associated genes resulted in pronounced viability and/or cell migration defects in human lung cancer cells. Progression-associated genes moreover exhibited strong associations with overall survival, specifically in human lung adenocarcinoma, but not in squamous cell carcinoma. The KM mouse model faithfully recapitulates key molecular events in human adenocarcinoma of the lung and is a useful tool for mechanistic interrogation of KRAS-driven LuAd progression.

## 1. Introduction

Genetically engineered mouse models (GEMMs) are powerful tools for understanding the roles of specific oncogenes and tumour suppressors in cancer development and progression. The term “GEMM” spans a broad spectrum of design strategies, ranging from constitutive whole-body deletion of tumour suppressors or overexpression of oncogenes from tissue-specific promoter fragments, the latter typically resulting in artificially high levels of oncogene expression in every cell of a given tissue, to more refined conditional allelic models that allow for sporadic activation/deletion of the target allele in a temporally controlled manner [1,2]. For even the most elegantly designed GEMMs. However, there are valid concerns about the ability of such models to faithfully recapitulate the molecular evolution and features of human cancer [3]. This is particularly true for cancers, such as lung cancer, that arise primarily from exposure to environmental toxins. Given that GEMMs are increasingly used for pre-clinical testing of novel treatment strategies it is vitally important to interrogate their suitability for this role [4].

Development of the lsl-KRas^G12D^ allele, in which a single-nucleotide altered coding exon of KRas was inserted into the endogenous KRas locus downstream of a CRE-deletable lox-stop-lox cassette, represented a watershed moment in the progress of GEMM development [5]. Aside from the requirement for exposure to CRE recombinase, expression of this allele is entirely physiological, marking a major departure from previous transgenic models that typically relied on pronounced overexpression of oncogenes from strong tissue-restricted promoters [6]. An oncogene that is overexpressed as well as mutated will elicit a much stronger signal than one that is merely mutated, with profound implications for phenotypic evolution [7]. Given that KRAS is much more frequently mutated without overexpression than with [8], the lsl-KRas^G12D^ allele better reflects the expression of activated KRAS in human disease than would an overt overexpression allele. Perhaps unsurprisingly, this allele emerged to be relatively inefficient in driving late stage cancer but very effective in driving incipient early stage disease [5]. The modest oncogenic output of lsl-KRas^G12D^, however, turns out to be its principle strength, allowing for the investigation of spontaneous tumour progression from pre-cancerous lesions. Accordingly, combination of this allele with additional oncogenes or tumour suppressors readily drives progression of incipient tumours [9,10,11].

By comparison, modeling the oncogenic output of MYC deregulation at a level that reflects incipient human cancer is more challenging. Although MYC expression is typically quite high in late-stage human cancers, pronounced overexpression of MYC in normal tissue immediately elicits an apoptotic response, eliminating the offending cells [12,13,14]. The prior or simultaneously inactivation of apoptosis is typically a prerequisite for tumour initiation by high levels of MYC [15,16]. Insertion of a conditional and acutely inducible allele of c-MYC into the Rosa26 locus led to the serendipitous discovery of a proliferation competent level of MYC expression below the threshold required to drive apoptosis [17]: Rosa26-lsl-MycER^Tam^ mice express a Tamoxifen-dependent allele of the c-MYC oncogene fused in-frame to a minimal ligand-binding domain of human estrogen receptor, the latter modified to prevent activation by circulating estrogen [18]. Functional characterization of this allele demonstrated expression of MycER^Tam^ in the near-physiological range, relative to endogenously expressed c-Myc. Importantly, this level of expression sufficed to drive ectopic proliferation in all adult organs examined, without triggering widespread MYC-dependent apoptosis [17]. The implication of this observation is that modest MYC overexpression may, by virtue of evading intrinsic tumour suppression, exhibit enhanced tumour promoting activity compared with higher levels that overtly trigger apoptosis [19,20]. The sustained activity of MycER^Tam^, however requires continuous administration of the synthetic ligand Tamoxifen, which exerts both agonist and antagonist activity on cells expressing the endogenous estrogen receptor, potentially impacting on MYC-driven phenotypes [21]. We, therefore, re-engineered the allele without the ER moiety to retain the low level of expression afforded by the Rosa26 locus and the CRE-dependence of expression, but to liberate MYC’s transcriptional activity from its dependence upon Tamoxifen (Figure S1). By combining the Rosa26-DM.lsl-MYC allele with lsl-KRas^G12D^, we aimed to model tumour evolution in an important subset of human non-small cell lung cancers. Here, we show accelerated development of low-grade adenocarcinomas that spontaneously progress to locally invasive disease. Transcriptomic analysis of spontaneously progressing tumours identified a gene expression signature of clear relevance for human lung adenocarcinoma.

## 2. Results

### 2.1. MYC Accelerates KRas^G12D^-Driven LuAd Development

**Figure 1:**
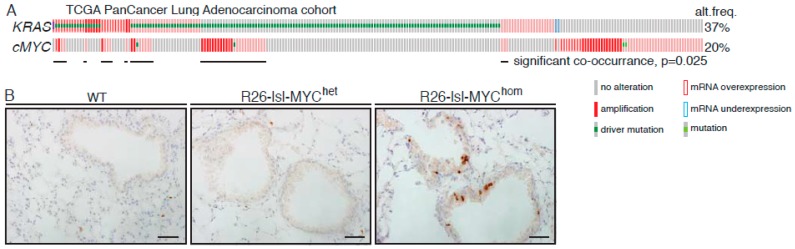

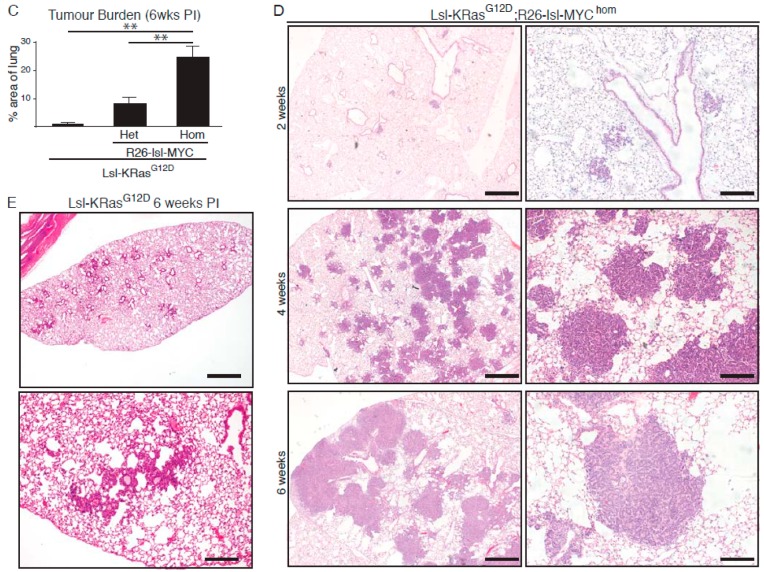
MYC accelerates KRas^G12D^-driven lung adenocarcinoma (LuAd) development. (**A**) Frequency of mutation, copy number, and mRNA expression alteration of KRAS and cMYC in the TCGA pan cancer lung adenocarcinoma cohort, accessed through cBioportal. For mRNA analysis, Z score threshold was set to 1.5. Horizontal black lines indicate cases with alteration of both KRAS and MYC. (**B**) Ectopic proliferation induced by CRE-dependent activation of Rosa26-lsl-MYC in airway epithelium evidenced by BrdU incorporation. Images are representative of at least 4 mice/genotype. Scale bar = 40 μm. (**C**) Overall tumour burden, defined as the percent of lung area occupied by tumour tissue, in mice bearing 1 (*N* = 9) or 2 (*N* = 11) R26-lsl-MYC combined with lsl-KRas^G12D^, compared with lsl-KRas^G12D^ alone (*N* = 6), measured 6 weeks post induction (PI). Mean ± SEM shown. ** denotes *p* < 0.01 (*t*-Test). (**D**) Adult mice (8–10weeks old) bearing the indicated conditional alleles were administered 1 × 10^7^ pfu Adeno-CRE by intranasal installation and harvested at the indicated times PI. Micrographs show representative Hematoxylin and Eosin (H&E)-stained lung tissue. Scale bars = 1mm (left panels) and 200 μm (right panels). (**E**) Age-matched lsl-KRas^G12D^ mice were induced with the same pfu Adeno-CRE as (D) and tumour burden examined after 6 weeks. Scale bars = 1mm (top panel) and 200 μm (bottom panel).

Analysis of the TCGA (the cancer genome atlas) pan-cancer cohort of lung adenocarcinoma (LuAd) revealed amplification and/or overexpression of *c-MYC* in up to 20% of LuAd and a significant enrichment for *MYC* overexpression in *KRAS* mutant tumours (*p* = 0.025) (Figure 1A and Figure S2). We used replication defective recombinant adenoviral delivery of CRE recombinase (Ad-CRE), administered by intranasal inhalation, to sporadically activate expression of transgenic MYC in the lungs of heterozygous Rosa26^DM.lsl-MYC/+^ (M) and homozygous Rosa26^DM.lsl-MYC/lsl-MYC^ (M^2^) mice. Acute ectopic proliferation of airway epithelium, detected by BrdU incorporation 3 days after allele activation, was only detectable in homozygous mice (Figure 1B). No tumours could be detected in Ad-CRE induced M or M^2^ mice housed for up to 1 year after induction [22]. We, therefore, asked if Rosa26-driven MYC could cooperate with endogenously expressed active KRas to accelerate lung tumour development. Comparison of the lung tumour burden in Ad-CRE induced lsl-KRas^G12D/+^ (K), lsl-KRas^G12D/+^; Rosa26^DM.lsl-MYC/+^ (KM), and lsl-KRas^G12D/+^; Rosa26^DM.lsl-MYC/lsl-MYC^ (KM^2^), mice at 6 weeks post induction (PI) revealed a dramatic, MYC-dose dependent, increase in the percentage of lung area occupied by tumours (Figure 1C). Histopathological analysis of KM^2^ tumours at 2, 4, and 6 weeks showed uniform progression of all incipient KM^2^ tumours to low-grade (non-invasive) adenocarcinoma in situ (Figure 1D), as previously defined [23]. This contrasts with KRas^G12D/+^-only lesions that fail to progress beyond atypical adenomatous hyperplasia within this time (Figure 1E) [5].

### 2.2. A Transcriptomic Signature of KRAS LuAd Tumour Progression

As we recently reported [24], KM^2^ tumours sporadically progress to a higher-grade disease characterized by increased morphologic heterogeneity and sharply increased phosphorylation of Erk1/2, downstream of KRAS (Figure S3). We used laser-capture microdissection to collect such p-Erk^HI^ tumour regions, along with matching p-Erk^LO^ regions from the same tumours, and RNA-SEQ analysis to determine changes in gene expression associated with progression of KM^2^ LuAd [24]. Across tumours from four mice we detected 1396 genes with significantly altered expression. To establish an expression signature of general relevance to human cancer irrespective of KRAS status, we used an arbitrary cutoff of 2.5X increased expression and evidence of overexpression and/or amplification from human LuAd datasets [25,26,27] available via Oncomine and cBioportal to stratify significantly upregulated genes, yielding a list of 52 genes (Table 1). This list notably includes EGFR/ERBB-family ligands *Ereg* and *Areg*, Wnt pathway constituents *Sox9* and *Porcn*, nutrient transporters *Slc2a1* and *Slc38a1*, and glycolytic enzymes *Gapdh* and *Pgk1*, the latter four suggestive of increased metabolic demand during LuAd progression.

### 2.3. Functional Validation of p-Erk Associated Genes: Contribution to Cell Propagation

**Figure 2:**
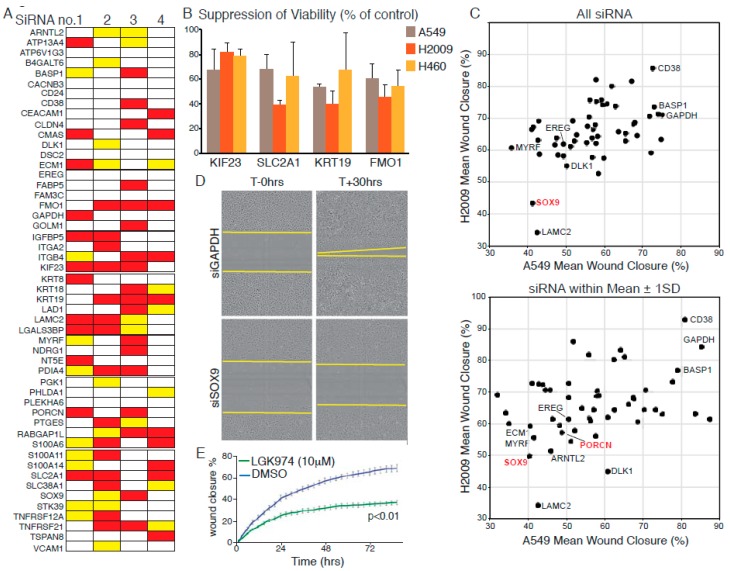
Validation of KM tumour progression signature genes in human cell lines.(**A**) Summary of suppression of viability (SoV) screening data using four individual siRNAs to target each indicated gene in A549, H460, and H2009 cells. Red bars indicate greater than average SoV in all three cell lines; Yellow bars indicate greater than average SoV in 2/3 lines. (**B**) Mean (±SD) SoV of three siRNAs, targeting each of the indicated transcripts, that exhibit consistent effects in all three cell lines. (**C**) Suppression of H2009 (*y*-axis) and A549 (*x*-axis) cell migration upon depletion of gene products listed in (**A**). Top panel: Mean distance migrated (defined as percent “wound” closure) after separate transfection with four siRNAs targeting each gene. Lower panel: Mean migration distance values for siRNAs falling within one standard deviation of the mean of all four for each targeted gene. (**D**) Representative photomicrographs showing suppression of cell migration upon depletion of Wnt pathway component SOX9. Compare with GAPDH (top panels). (**E**) Pharmacological suppression of Wnt signalling using the PORCN inhibitor LGK974 suppresses migration of A549 cells, as determined by Incucyte time-lapse microscopy. Error bars show SD of technical triplicates. *p* value from *t*-Test shown. All data are representative of three or more experiments.

We assembled a focused library of four separate siRNAs targeting each of the 52 p-Erk^HI^-associated genes implicated in human non-small cell lung cancer (NSCLC) and confirmed the efficacy of targeted depletion of a randomly chosen subset in A549 cells using Quantitative-PCR (Figure S4A). We then screened the effect of each siRNA individually for the ability to suppress propagation of 3 KRAS-mutated human NSCLC cell lines, A549 (G12S), H2009 (G12A), and H460 (Q61H). Cells were reverse transfected with individual siRNAs at low density and allowed to propagate for up to 72 h, whereupon, cell numbers were measured by high-content microscopy. Each screen included positive (Allstars, Qiagen, Hilden, Germany) and negative (non-targeting siRNA) controls. The effect of each individual siRNA on cell propagation was calculated as percent suppression of viability (SoV), relative to non-targeting siRNA-transfected cells, in each of the three cell lines. The mean SoV of all siRNAs across the entire dataset (M) was calculated for each cell line and used to set a threshold for acceptance or rejection of the effects of individual siRNAs: siRNAs that resulted in SoV > M were, thus, scored “positive”. Figure 2A summarizes the results of this analysis: siRNAs yielding greater than average SoV in all three cell lines are indicated by red bars; those that scored positive in 2/3 cell lines by yellow bars. Thus, for each *FMO1*, *KIF23*, *KRT19,* and *SLC2A1*, the same three out of four siRNAs consistently suppressed cell propagation in all three human NSCLC lines tested (Figure S4B) allowing us to say with confidence that these four genes are required to sustain proliferation of KRAS mutant human NSCLC cells. The functional requirement of nascent lung tumours for Kif23 has moreover been verified in vivo [27].

### 2.4. Functional Validation of p-Erk Associated Genes: WNT Signalling Contributes to Cell Motility

Given the association of the dataset with an invasive phenotype [23] we used our focused siRNA library to ask if any of the selected genes affect migration of human NSCLC cells. RNAi reverse transfected A549 or H2009 cells were seeded in 96-well plates and grown to confluency over 48 h. Monolayers were then scratch ‘wounded’ with a woundmaker tool and monitored by Incucyte time-lapse video microscopy until the scratch wounds of control siRNA-transfected cells were circa 90% closed. Total cell number per well was determined at the end of each experiment to assess the potential influence of loss of viability (LoV) upon the migration assay. The mean wound closure and LoV values were determined for each siRNA and averaged across all four siRNAs for each gene. Figure S2B shows the mean migration data plotted against the mean LoV for each gene for A549 and H2009 cells (H460 cells were omitted from this analysis as they failed to migrate under the conditions tested). Only in the case of KIF23 depletion was LoV found to consistently account for the observed suppression of scratch wound closure. Depletion of LAMC2, previously validated as required for invasion and metastasis in NSCLC [28], consistently suppressed migration in both cell lines. Interestingly, depletion of WNT signalling components, SOX9 or PORCN [29], also suppressed migration in both cell lines (Figure 2C,D), suggesting that WNT signalling contributes to NSCLC tumour progression beyond its ability to suppress senescence [30,31,32]. This effect was confirmed in A549 cells by pharmacological inhibition of PORCN (Figure 2E).

### 2.5. Relevance of the Murine Dataset for Human Pulmonary Adenocarcinoma

**Figure 3:**
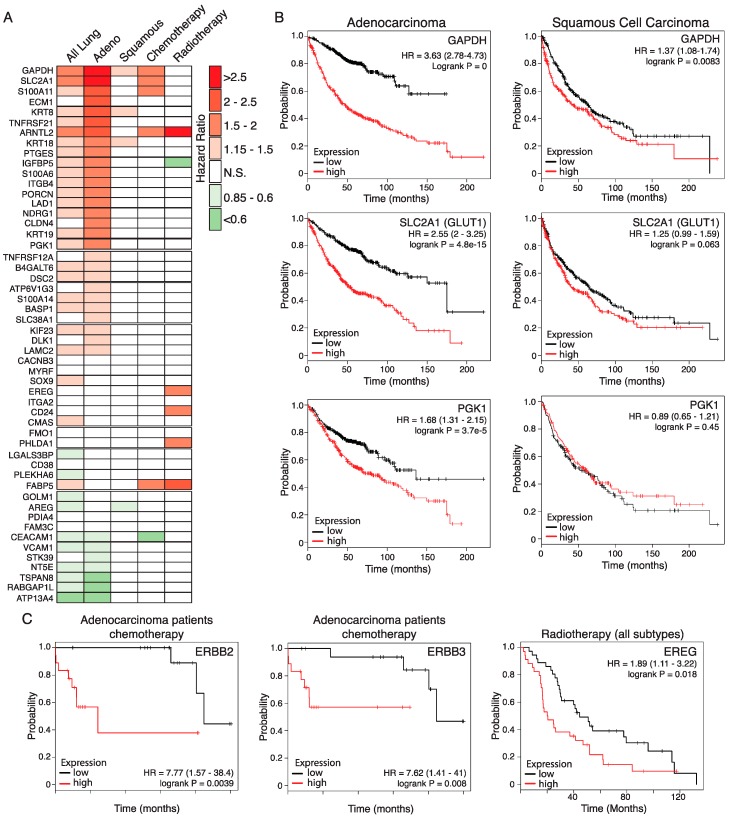
Relevance of the KM tumour progression signature for overall survival of human LuAd patients. (**A**) Hazard ratios (HRs) of lung cancer patient overall survival associated with high expression of genes listed in Table 1, determined on the basis of above-versus-below median gene expression, using the KMplot survival analysis portal. Analysis shows statistically significant HRs for all lung patients included (*N* = 1926), those with denocarcinoma (Adeno; *N* = 866), squamous cell carcinoma (Squamous; *N* = 675), and patients who received either chemotherapy (*N* = 178) or radiotherapy (*N* = 73). N.S. = Not statistically significant. (**B**) Overall survival plots based on above (red lines) versus below (black lines) median expression of glycolysis pathway genes in human lung adenocarcinoma (left panels) compared with lung squamous cell carcinoma (right panels). HR = hazard ratio. 95% confidence intervals shown in parenthesis. For the adenocarcinoma subtype, N = 866; for the squamous subtype, *N* = 675. (**C**) High expression of ERBB2 and ERBB3 is associated with worse outcome in human adenocarcinoma patients receiving standard chemotherapy. Note that the low sample size available for this subgroup (*N* = 36) require that the data be considered preliminary. Right panel: High expression of the ERBB ligand EREG is associated with worse outcome for lung cancer patients receiving radiotherapy (*N* = 73). Low sample size precluded analysis of histological subtypes.

We used an online Kaplan Meier meta-analysis tool [28] to investigate the association of each of the 52 selected genes with overall survival (OS) and responsiveness to radio- or chemotherapy among human lung cancer patients. Cox regression plots of patient survival were generated based on a median split of gene expression levels above (High) or below (Low) median gene expression. Differential expression of 33 of the 52 genes is associated with significantly altered OS across all lung cancers (*N* = 1926), with high expression of 25 genes associated with significantly decreased OS (logrank *p* < 0.05). Upon analysis of histological subtypes, 35 genes are associated with significantly altered OS of adenocarcinoma patients (*N* = 719), with 28 genes overlapping with those significantly altered across all lung tumours. In contrast, only three genes (*KRT8, KRT18,* and *GAPDH*) correlate with significantly reduced OS of squamous cell carcinoma patients (*N* = 525), mirroring the histological classification of the murine KM tumours as adenocarcinoma (Figure 3A). Strikingly, high expression of *SLC2A1*, encoding the glucose transporter GLUT1, and the glycolytic genes *GAPDH* and *PGK1*, were associated with strongly reduced OS, particularly in the adenocarcinoma subtype (Figure 3B), consistent with the prognostic value of FDG-PET in LuAd [29].

Across all lung cancers, high expression of five of the 52 genes is associated with significantly worse outcome in patients who received chemotherapy (*N* = 176): *SLC2A1, S100A11, GAPDH, FABP5,* and *ARNTL2*. The latter two are also associated with worse outcomes in patients who received radiotherapy (*N* = 70), with hazard ratios of 2.34 and 3.25, respectively. High expression of a further four genes is also associated with poorer response to radiotherapy: *S100A14, PHLDA1, EREG,* and *CD24* (HRs ranging from 1.89 to 1.93). In contrast, high *IGFBP5* expression is associated with a better outcome in patients who received radiotherapy (HR = 0.56) while high *CEACAM1* expression is associated with a better outcome in patients who received chemotherapy (HR = 0.57). Expression of ERBB ligands *EREG* and *AREG* previously led us to identify an unexpected requirement for signalling through the ERBB family of receptor tyrosine kinases for development, progression, and maintenance of KRAS driven LuAd, verified independently by a second group [24,30]. Notably, high expression of either *ERBB2* or *ERBB3* is associated with resistance of adenocarcinoma to chemotherapy, although these data should be interpreted with caution due to the low sample sizes available (Figure 3C). The KM^2^ mouse model, thus, exposes a number of genes that have a meaningful impact on the overall survival and response to therapy of human lung adenocarcinoma, attesting to its value as a model for this disease in humans.

## 3. Discussion

We previously identified a gene signature associated with sporadic progression to locally invasive adenocarcinoma in a GEMM of lung cancer driven by endogenously expressed KRas^G12D^ combined with modestly overexpressed c-MYC [24]. Systematic analysis of each individual gene in this signature in multiple human NSCLC cell lines has identified conserved requirements for cell proliferation and identified a key role for WNT signalling in NSCLC cell migration. Crucially, analysis of human lung cancer patient survival data demonstrates that the KM^2^ progression signature encompasses many genes that are significantly and specifically associated with the overall survival of lung adenocarcinoma patients. It should be noted that the patient survival analysis made no reference to KRAS mutation status and, thus, includes patient with EGFR, ALK, and other undefined driver mutations. The nevertheless strong association of the signature with the adenocarcinoma subtype may, thus, reflect general properties of LuAd tumour progression, irrespective of the driver oncogenes present. Alternatively, further stratification by driver oncogene may enhance some of these associations. Intriguingly, several signature genes are associated with differential survival of patients treated with chemotherapy versus radiotherapy. Further validation of such genes may, in future, help guide selective treatment strategies for LuAd.

The KM^2^ spontaneous progression signature highlights the specific impact of the glycolytic cascade on patient survival: Elevated expression of the glucose transporter *SLC2A1* and glycolytic enzymes *GAPDGH* and *PGK1* are associated with profoundly lower overall survival of denocarcinoma patients, with more modest associations in the squamous cell subtype. Increased glycolytic activity linked to tumour progression was also observed in an independent GEMM model and correlated with the amplification of the active KRas^G12D^ allele [31]. As well as being processed for ATP consumption and macromolecular synthesis, much glucose uptake is diverted via the pentose phosphate and 1-carbon pathways for maintenance of cellular anti-oxidant capacity [32]. Although not included in our progression-associated gene signature, we found increased expression of many anti-oxidant genes as KM^2^ tumour progress to invasive disease. The clinical relevance of anti-oxidant gene expression in LuAd is a reflected loss of function mutations in KEAP1, which suppresses anti-oxidant gene expression, in some 20% of cases. Genetic deletion of Keap1 or overexpression of its target, the anti-oxidant master regulatory transcription factor NRF2, accelerates KRas-driven LuAd in mice, suggesting that excessive oxidative stress limits tumour progression [33,34]. Targeted suppression of the anti-oxidant pathway may, thus, expose lung tumours to excessive oxidative stress, leading to improved therapeutic outcomes [35].

Along the same lines, the KM^2^ progression signature led us to identify an unexpected requirement for signalling through the ERBB family of receptor tyrosine kinases upstream of KRAS, despite the presence of the G12D activating mutation [24]. ERBB family proteins have proven to be far more amenable to pharmacological inhibition than KRAS itself, and multiple ERBB inhibitors are approved for the treatment of cancers expressing mutant or amplified ERBB isoforms. Indeed, we and others have demonstrated the efficacy of broad-spectrum ERBB inhibitors against KRAS driven LuAd in vitro and in vivo [24,30,36]. To this we now add patient survival data associated with overexpression of ERBB receptors and ligands, suggesting that elevated activity of this pathway may strongly influence responses to standard-of-care therapies. It is important to note that considerable redundancy exists within the ERBB network, between four receptors that can all homo- or hetero-dimerise upon binding of to up to 10 or more ligands. As such, broad specificity inhibition of all four RTKs may be more desirable than selective inhibition of any one [37].

The KM^2^ model, thus, recapitulates multiple salient molecular features with clear clinical relevance for human lung adenocarcinoma. Data presented here represent the analysis of the earliest detectable spontaneous progression of KM^2^ tumours to invasive heterogeneous disease, and it is worth noting that tumour heterogeneity increases along with the emergence of metastases upon longer incubation [24]. The model, thus, allows incipient tumours to evolve sporadically, driven by emerging selective pressures rather than by the immediate consequence of transgene expression. Allowing autochthonous tumours to evolve in this manner likely exposes them to many of the same physiological barriers encountered by incipient human tumours, despite the complete absence of environmental toxins in the GEMM system, and this is reflected in the tumour progression signature. We believe that the KM^2^ model is, thus, an excellent system for further investigation of lung tumour evolution and for pre-clinical evaluation of new therapeutic strategies.

## 4. Materials and Methods

### 4.1. Genetically Engineered Mice and Mouse Procedures

Procedures involving mice were performed in accordance with protocol numbers 55.2-2531.01-72/10 (University of Würzburg, Würzburg, Germany) and Home Office licence numbers 60/4183 and 70/7950 (CRUK BICR, Glasgow, UK). Rosa26^DM.lsl-MYC^ mice were generated as previously described [24] using vectors described in [38]: Targeted insertion into the *Rosa26* locus was confirmed by Southern blotting, and genotyping was performed using the following primers: A) CCC AAA GTC GCT CTG AGT TG (common); B) GCG AAG AGT TTG TCC TCA ACC (targeted locus); C) GGA GCG GGA GAA ATG GAT ATG A (wild-type locus). All genotyping was subsequently performed by Transnetyx Inc. *Lsl-KRas^G12D^* [5] mice were obtained from the NCI (US National Cancer Institute) mouse repository at Fredrick, MD, USA. Mice were maintained on a mixed FVBN/C57Bl6 background, housed on a 12 h light/dark cycle and fed and watered ad libitum. Both males and females were included in the study. Recombinant adenovirus expressing CRE was purchased from the University of Iowa gene therapy core facility. For Adeno-Cre installation, mice aged 8 weeks were sedated with a mixture of domitor and ketamine, injected IP. For most experiments, 1x10^7^ pfu Adeno-CRE were administered intranasally using the calcium phosphate precipitation method, as described previously [17]. BrdU injection was performed at least 2 h prior to sacrifice. All mice were sacrificed using a schedule 1 procedure. Rosa26^DM.lsl-MYC^ mice are available upon request from D.J.M.

### 4.2. Immunohistochemistry and Tissue Analysis

Mouse tissues were perfused with zinc formalin overnight. Four micrometer paraffin sections were de-paraffinized and rehydrated: 3 × 5 min xylene, 2 min in each 2 × 100%; 2 × 95%; 2 × 70%; 1 × 50% ethanol; dH_2_O. Peroxidase blocking was performed for 10 min in 3% H_2_O_2_ diluted in H_2_O, followed by antigen retrieval in 10 mM citrate buffer, pH 6, 10 min near boiling by microwave heating at low power. Non-specific antibody binding was blocked with up to 3% BSA or up to 5% normal goat serum for 1 h at RT or overnight at 4 °C. Primary antibody incubation was performed overnight at 4 °C or 2 h at 37 °C. Secondary biotinylated antibody was incubated 1 h at room temperature, and stain was developed with stable DAB (Diaminobanzadine, Invitrogen, Carlsbad, CA, USA) followed by counterstaining with Gil 1 hematoxylin (MH216, Sigma Chemicals, St. Louis, MI, USA) and Scotts tap water substitute. The following antibodies were used at the indicated dilution: p-Erk (P44/42 MAPK phosphor-Thr202/Tyr204), Cell Signalling CS4370, BrdU, Serotec OBT0030CX, 1:250 or BD Biosciences 347580 1:200; Vectorlabs VECTASTAIN ABC kit; anti-Rabbit IgG (immunoglobulin G), PK-4001 ECL (enhanced chemoluminescence reagent); anti Rat, GE Healthcare NA935. Tumour burden was determined using Leica software as the percentage area of lung tissue occupied by tumours, measured on three Hematoxylin and Eosin (H&E) stained sections, separated by at least 100 μm, from each of the indicated numbers of mice. Histological classification of tumours as adenocarcinoma was determined by a clinical pathologist. Laser-capture and RNA-SEQ analysis were performed as previously described [24]. RNA-SEQ data are available from Arrayexpress: E-MTAB 6432.

### 4.3. Cell Culture and Related Assays

Established lung cancer cell lines (A549, H2009, H460) were validated in-house and grown in DMEM (Dulbecco’s modified Eagle medium) with 10% FBS and penn/strep. A focused de-convoluted library of 4 siRNAs targeting each of 51 selected gene products was purchased from Qiagen. For analysis of cell viability, cells were reverse transfected with individual siRNAs in 96-well plates at a density that yielded 70% confluence after 72 h incubation when transfected with non-targeting control siRNA. Cells were transfected with each siRNA in technical quadruplicate, using either Lipofectamine 2000 or RNAi Max, after optimization of transfection for each cell line. Seventy-two hours later, cells were washed with PBS (phosphate-buffered saline), fixed with 4% PFA (paraformaldehyde) for 20 min, stained with DAPI (4′,6-diamidino-2-phenylindole) and nuclei were counted using Columbus Image Analysis following image acquisition on an Operetta High Content Analyzer. Screening was performed in triplicate for each cell line. To measure effects on cell migration, cells were reverse transfected in 96-well plates at a density that yielded 100% confluence after 48 h when transfected with non-targeting siRNA. After 48 h, scratch wounds were made using a 96-point woundmaker tool. Scratch wounds were measured by Incucyte time-lapse video microscopy for up to 46 h (A549) or 20 h (H2009) after wounding. Cells were then fixed and DAPI-stained nuclei counted by Operetta. Migration screens were performed in triplicate for each cell line.

### 4.4. Statistical Analysis

Analysis was performed as previously reported [24]: Raw data obtained from quantitative real time PCR (polymerase chain reaction), FACS (fluorescence activated cell sorting), siRNA (small inhibitory ribonucleic acid) screening and Incucyte assays were copied into Excel (Microsoft, Redmond, WA, USA) spreadsheets. All Mean values, SD, and SEM values of biological replicates were calculated using the calculator function. Graphical representation of such data was also produced in Excel (Microsoft). Statistical significance for pairwise data was determined by the Student’s *t*-Test. For multiple comparisons, ANOVA was used with a post-hoc Turkey test. * denotes *p* < 0.05; ** denotes *p* < 0.01; *** denotes *p* < 0.005. For analysis of human survival data, univariate Cox regression plots to assess the association of individual genes with lung cancer patient overall survival were plotted using KMplot.com [28] with a median patient split. Where multiple probes were available for a single gene, the median hazard ratio is presented. *p* values from Logrank tests are provided.

## 5. Conclusions

A gene expression signature associated with spontaneous progression of murine lung tumours driven by the combination KRas^G12D^ and modest overexpression of MYC identified multiple genes required for viability and/or cell migration of KRAS mutant human lung tumour cells. In vitro validation of individual genes exposed an unexpected requirement for Wnt signalling in cell migration. Analysis of human lung cancer patient survival revealed the significant prognostic association of individual genes, specifically for the adenocarcinoma subtype, along with several potential predictors of patient responses to standard chemo or radiotherapy.

## Figures and Tables

**Table 1:** Specific genes upregulated in pErk^High^ KM tumour regions. Genes were selected based on evidence of overexpression and/or amplification in human non-small cell lung cancer (NSCLC). Range of RNA-SEQ read count values, normalised for total reads per sample, for the indicated genes. FC = fold change. FDR = false discovery rate. Amp = Amplified. OverEx = Overexpressed. For “Amplified in human NSCLC”: Y = yes (1–5%) with >5% and <1% where noted; N = No; SCC = squamous cell carcinoma where noted, otherwise adenocarcinoma or unclassified. For “Overexpressed in human NSCLC”: Y = Yes; N = No.

Symbol	Gene Name	Range p-ERK neg	Range p-ERK pos	Mean FC	FDR	Amp in NSCLC	OverEx in NSCLC
Ereg	Epiregulin	5–39	227–376	24.82	2.78e-13	Y	Y
Sox9	SRY-like containing gene 9	6–31	39–330	15.79	7.48e-04	Y	Y
Dlk1	Delta-like 1 homolog	63–400	341–2114	10.46	2.33e-26	Y(SCC)	N
B4galt6	UDP-Gal:betaGlcNAc beta 1,4-galactosyltransferase, 6	7–91	113–491	9.46	1.24e-03	Y(SCC)	Y
Basp1	Brain abundant, membrane attached signal protein 1	0–66	89–307	9.03	1.31e-04	Y > 10%	Y
Itga2	Integrin alpha 2	49–185	185–1239	6.79	2.47e-11	<1%	Y
Cldn4	Claudin 4	7–49	64–117	6.11	1.16e-02	Y	Y
CD24a	CD24a antigen	145–1040	1411–1580	5.94	1.4e-15	No data	Y
Slc38A1	Solute carrier family 38, member 1	60–153	248–716	5.68	1.17e-11	Y	Y
Arntl2	Aryl hydrocarbon receptor nuclear translocator-like 2	15–59	44–156	4.54	4.9e-02	Y > 7%	Y
Dsc2	Desmocollin	14–148	62–302	4.43	4.51e-02	Y(SCC)	Y
Areg	Amphiregulin	156–234	526–831	4.25	4.98e-11	Y	Y
S100a6	S100 calcium binding protein A6	115–215	234–1017	4.00	1.51e-07	Y > 14%	Y
Itgb4	Integrin beta 4	31–89	81–310	3.92	2.29e-02	Y	Y
Atp13a4	ATPase type 13A4	242–399	711–1002	3.92	3.03e-14	Y > 25%	Y
Kif23	Kinesin family member 23	151–359	313–994	3.76	8.81e-07	<1%	Y
Porcn	Porcupine homolog	38–52	76–182	3.59	4.85e-03	<1%	N
Krt8	Keratin 8	67–380	489–699	3.57	1.87e-05	Y	Y
Vcam1	Vascular cell adhesion molecule 1	11–133	75–442	3.57	3.59e-02	<1%	N
Krt18	Keratin 18	503–827	1192–1850	3.50	1.8e-11	Y	Y
Lamc2	Laminin gamma 2	457–677	1104–1825	3.48	2.56e-09	Y > 7%	Y
Tnfrsf12a	Tumour necrosis factor receptor superfamily, 12a	30–54	86–135	3.42	1.47e-02	Y	Y
Tspan8	Tetraspanin 8	329–570	635–1848	3.41	5.23e-06	Y > 5%	Y
Atp6v1g3	ATPase H+ transporting, lysosomal V1 subunit g3	46–94	91–232	3.40	2.8e-03	Y > 7%	N
Ecm1	Extracellular matrix protein 1	44–79	100–173	3.25	7.13e-03	Y > 16%	Y
S100a14	S100 calcium binding protein A14	89–210	218-530	3.18	6.52e-04	Y > 14%	Y
Lgals3bp	Lectin, galactoside-binding, soluble, 3 binding protein	178–333	472–679	3.16	2.09e-05	Y	Y
Nt5e	5’ nucleotidase, ecto	148–339	462–697	3.04	1.2e-05	Y(SCC)	Y
Lad1	Ladinin 1	141–344	401–619	3.01	2.92e-05	Y > 7%	Y
Igfbp5	Insulin-like growth factor binding protein 5	296–422	414–1197	2.99	1.36e-05	<1%	Y
Pdia4	Protein disulphide isomerase associated 4	966–1887	2519–3183	2.98	3.16e-05	Y	Y
CD38	CD38 antigen	37–64	77–167	2.97	3.82e-02	<1%	Y
Krt19	Keratin 19	203–469	664–969	2.96	2.06e-06	<1%	Y
Slc2a1	Solute carrier family 2, member 1	57–114	125–249	2.95	1.11e-02	Y	Y
Myrf	Myelin regulatory factor	57–163	95–474	2.92	1.26e-02	<1%	Y
Plekha6	Pleckstrin homology domain containing, family A, 6	185–353	490–692	2.91	3.34e-05	<1%	Y
Ndrg1	N-MYC downstream regulated gene 1	77–333	208–716	2.84	7.71e-03	Y > 5%	Y
Rabgap1l	RAB GTPase activating protein 1-like	1211–1754	2578–4099	2.83	3.95e-05	Y > 8%	N
Phlda1	Pleckstrin homology-like domain family A, member 1	105–226	175–402	2.82	7.01e-04	<1%	Y
Gapdh	Glyceraldehyde-3-phosphate dehydrogenase	79–106	136–216	2.80	1.08e-02	Y(SCC)	Y
Fabp5	Fatty acid binding protein 5, epidermal	78-175	115–410	2.79	6.15e-03	Y	Y
Cmas	Cytidine monophospho-N-acetylneuraminic acid synthetase	49–106	108–283	2.75	4.21e-02	Y > 5%	Y
Golm1	Golgi membrane protein 1	509–1420	1343–2171	2.75	3.84e-05	N	Y
Ptges	Prostaglandin E synthase	158–286	158–286	2.73	5.26e-03	Y	Y
Stk39	Serine/threonine kinase 39	304–659	872–1127	2.71	2.21e-06	Y	Y
Fam3c	Family with sequence similarity 3, member C	351–642	673–1078	2.65	6.4e-09	Y	N
Pgk1	Phosphoglycerate kinase 1	138–178	220–425	2.61	1.23e-03	<1%	Y
Cacnb3	Calcium channel, voltage dependent, beta 3	56–87	78–147	2.59	3.7e-02	Y	N
Tnfrsf21	Tumour necrosis factor receptor superfamily, 21	121–238	232–348	2.57	1.58e-03	Y	Y
Fmo1	Flavin containing monooxygenase 1	174–256	186–1098	2.57	7.51e-03	Y > 7%	Y
S100a11	S100 calcium binding protein A11	245–696	594–1252	2.53	1.21e-04	Y > 15%	Y
Ceacam1	Carcinoembryonic antigen-related cell adhesion molecule 1	326–608	648–1286	2.50	1.08e-05	Y	Y

## Supplementary Materials

The following are available online at https://www.mdpi.com/2072-6694/11/5/600/s1: Figure S1: Generation of Rosa26^DM.lsl-MYC^ mice. Figure S2: Statistical analysis of KRAS and MYC alterations in human LuAd. Figure S3: High-resolution images of K-only and KM^2^ tumour histology. Figure S4: In vitro validation of KM^2^ LuAd progression signature genes.
