# Supplementary material for: Identification of a Clinically Relevant Signature for Early Progression in KRAS-Driven Lung Adenocarcinoma
Source: Cancers (Basel). 2019 Apr 29;11(5):600. doi: 10.3390/cancers11050600 (PMC6562816; doi:10.3390/cancers11050600)
Supplement: Supplementary file 1 [file cancers-11-00600-s001.pdf]

# Supplementary Materials: Identification of a Clinically Relevant Signature for Early Progression in KRAS-Driven Lung Adenocarcinoma

**Sarah Neidler, Björn Kruspig, Kay Hewit, Tiziana Monteverde, Katarina Gyuraszova, Attila Braun[4], William Clark, Daniel James, Ann Hedley, Bernhard Nieswandt, Emma Shanks, Craig Dick and Daniel J. Murphy**

Figure S1

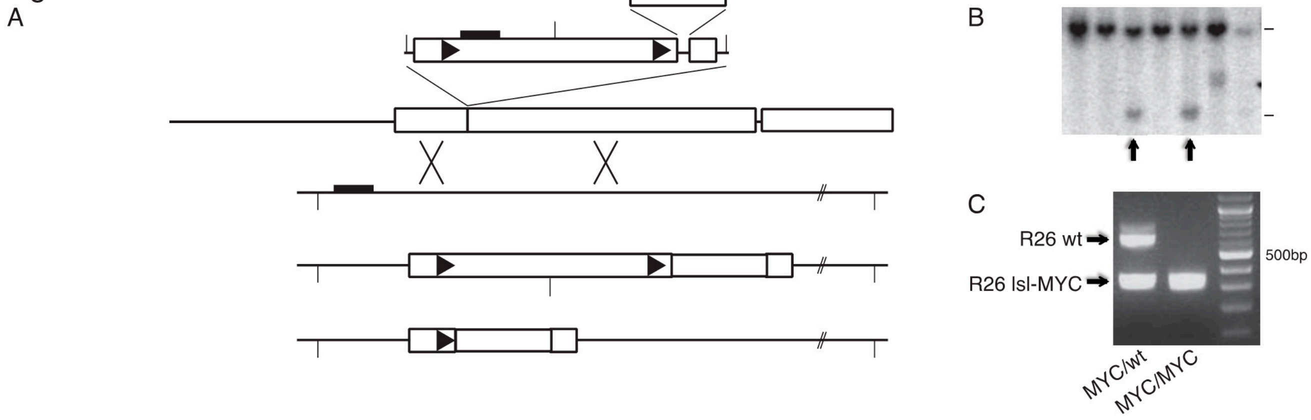


**Figure S1.** Generation and characterization of R26-lsl-MYC mice. (**A**) Schematic of Rosa26 targeting strategy adapted from Srinivas using vectors described by same [1]. (**B**) Southern blot of EcoRV-digested ES cell genomic DNA probed with external probe E. WT band = 11Kb; recombined band = 3.8Kb. Arrows indicate clones with the correctly targeted locus. (**C**) PCR of genomic DNA from heterozygous and homozygous R26-lsl-MYC mice.

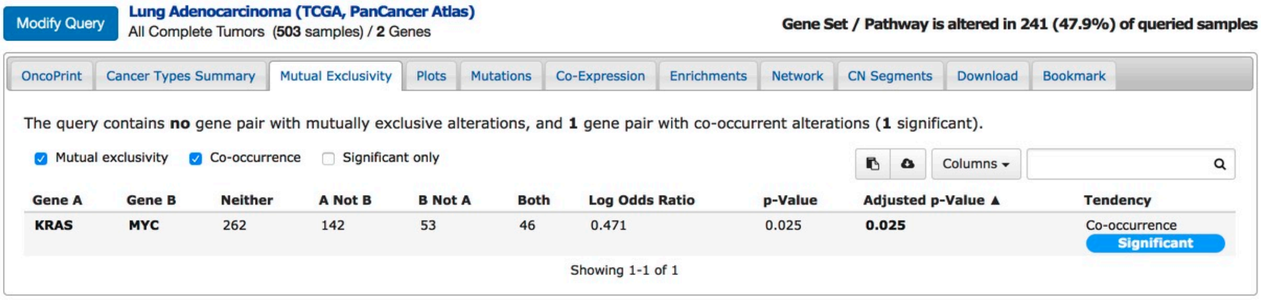


**Figure S2.** Significant co-occurrence of KRAS & cMYC alterations in human LuAd. Screenshot of cBioportal analysis of co-occurrent versus mutual exclusivity in alterations of KRAS and c-MYC in the TCGA PanCancer cohort of human LuAd.

Figure S3

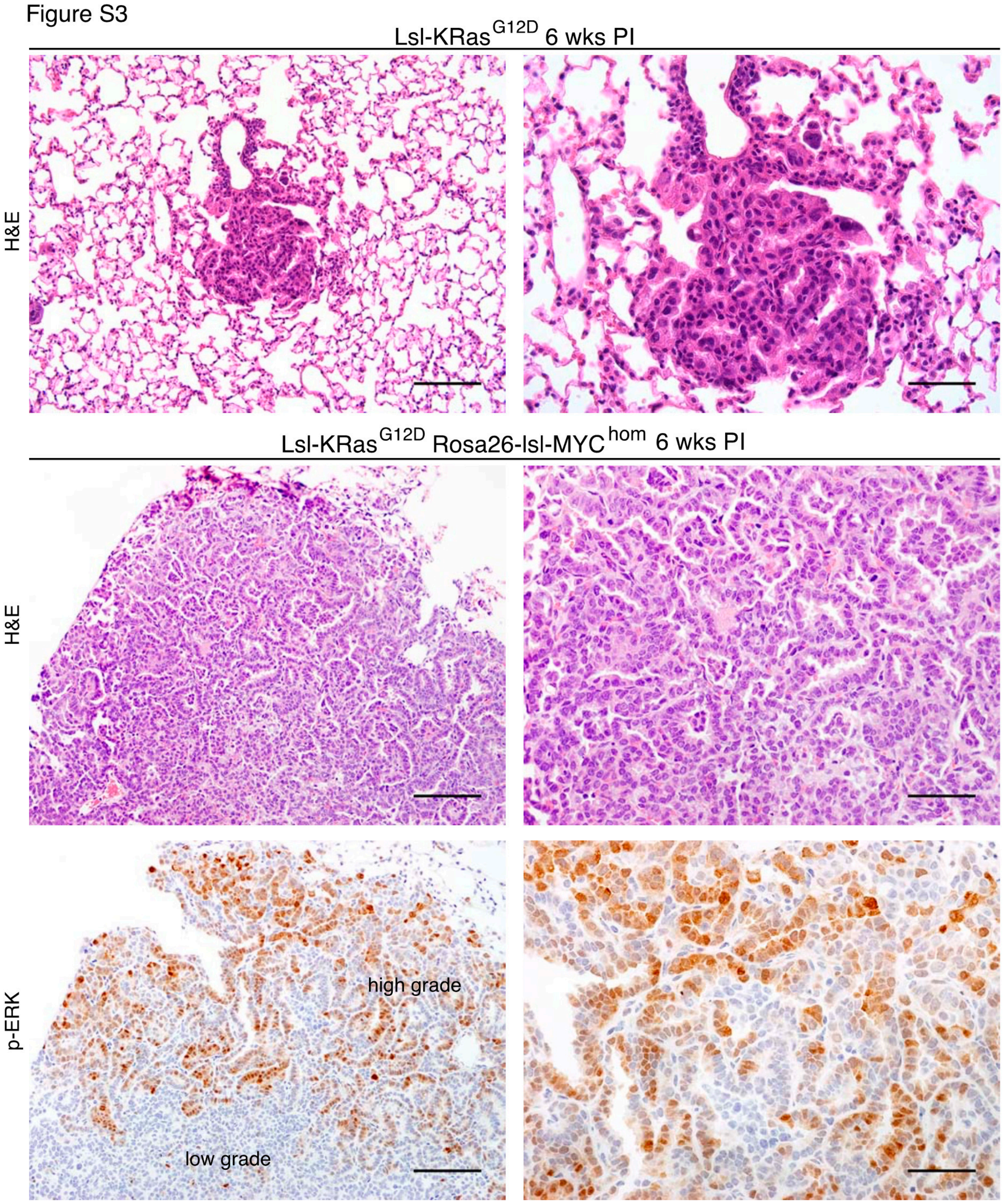


**Figure S3. Histology of K-only and KM² lung tumours at 6 weeks post induction.** Lung tumours were generated as per main Figure 1 and harvested at 6 weeks post induction. Top panels show atypical adenomatous hyperplasia in a K-only mouse. Middle and bottom panels show adenocarcinoma in a KM² mouse. Bottom panels show immunohistochemistry for phospho-ERK1/2, demarcating spontaneous progression from low grade to high grade adenocarcinoma. Scale bars = 50 µM (left panels) & 100 µM (right panels).

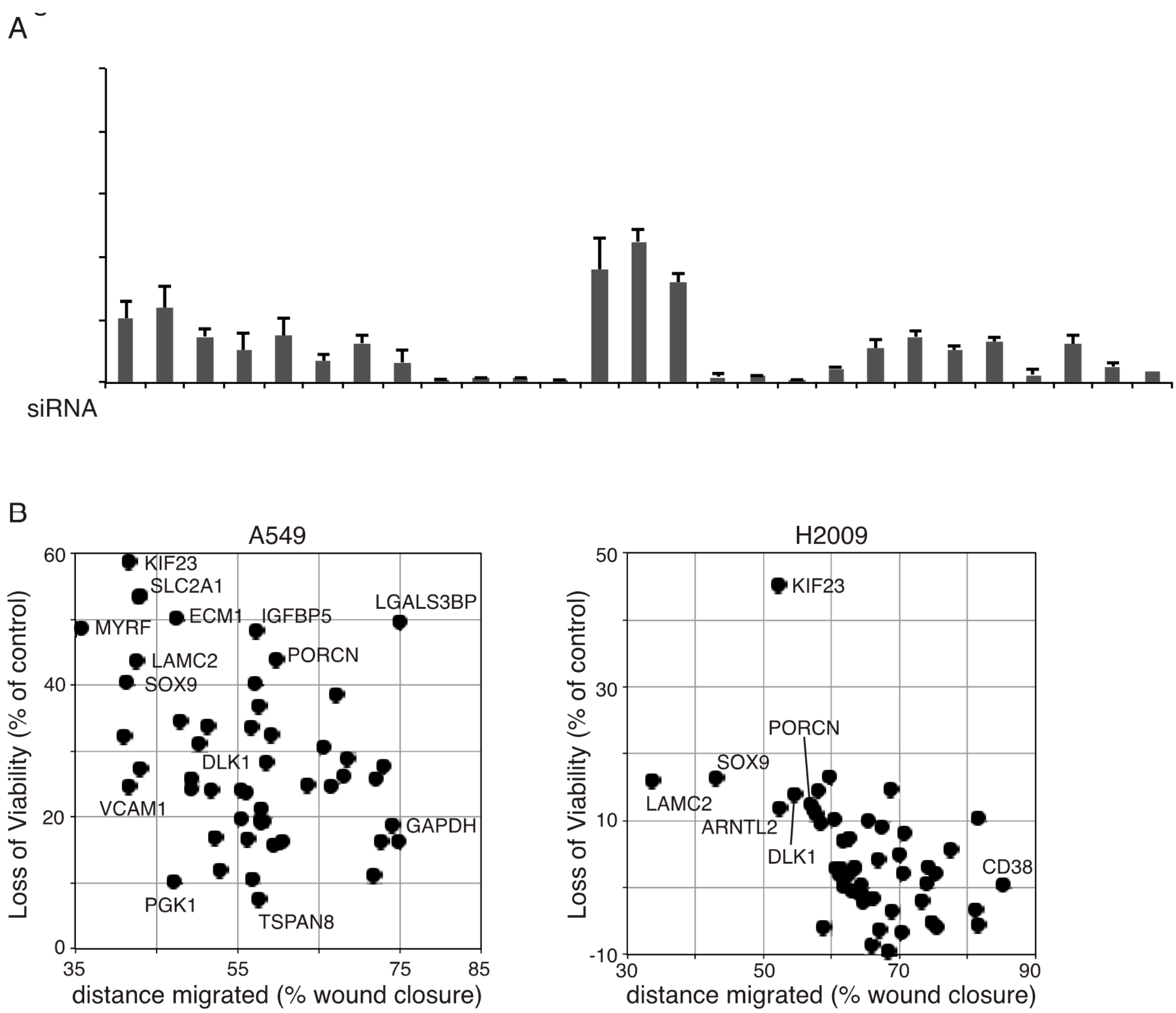


**Figure S4.** In vitro validation of KM tumour progression signature. (**A**) Confirmation of depletion of the indicated genes with individual siRNAs, performed by Q-PCR. Mean ± SEM of biological triplicates shown. (**B**) Loss of viability (*y*-axis) upon depletion of most targeted genes does not alone account for suppression of cell migration (*x*-axis). Mean values for 4 siRNAs targeting each gene shown. .

## Reference

1. Mishra, R.; Hanker, A.B.; Garrett, J.T. Genomic alterations of ERBB receptors in cancer: Clinical implications. *Oncotarget* **2017**, *8*, 114371–114392. doi:10.18632/oncotarget.22825.

© 2019 by the authors. Submitted for possible open access publication under the terms and conditions of the Creative Commons Attribution (CC BY) license (http://creativecommons.org/licenses/by/4.0/).
